# 3′RNA Sequencing Accurately Classifies Formalin-Fixed Paraffin-Embedded Uterine Leiomyomas

**DOI:** 10.3390/cancers12123839

**Published:** 2020-12-19

**Authors:** Miika Mehine, Sara Khamaiseh, Terhi Ahvenainen, Tuomas Heikkinen, Anna Äyräväinen, Päivi Pakarinen, Päivi Härkki, Annukka Pasanen, Ralf Bützow, Pia Vahteristo

**Affiliations:** 1 Applied Tumor Genomics Research Program, University of Helsinki, 00014 Helsinki, Finland; miika.mehine@helsinki.fi (M.M.); sara.khamaiseh@helsinki.fi (S.K.); terhi.ahvenainen@helsinki.fi (T.A.); tuomas.heikkinen@gmail.com (T.H.); anna.ayravainen@helsinki.fi (A.Ä.); annukka.pasanen@hus.fi (A.P.); ralf.butzow@hus.fi (R.B.); 2 Department of Medical and Clinical Genetics, University of Helsinki, 00014 Helsinki, Finland; 3 iCAN Digital Precision Cancer Medicine Flagship, 00014 Helsinki, Finland; 4 Department of Obstetrics and Gynecology, Helsinki University Hospital and University of Helsinki, 00029 Helsinki, Finland; paivi.pakarinen@eiransairaala.fi (P.P.); paivi.harkki@hus.fi (P.H.); 5 Department of Pathology, University of Helsinki and HUSLAB, Helsinki University Hospital, 00029 Helsinki, Finland

**Keywords:** 3′RNA sequencing, QuantSeq, fibroids, FFPE, *MED12*, *HMGA2*, *FH*

## Abstract

**Simple Summary:**

Uterine leiomyomas are benign smooth muscle tumors affecting millions of women globally. On a molecular level, leiomyomas can be classified into three main subtypes, each characterized by mutations affecting either *MED12*, *HMGA2*, or *FH*. Leiomyomas are still widely regarded as a single entity, although early observations suggest that different subtypes behave differently, in terms of both clinical outcomes and therapeutic requirements. The majority of classification studies on leiomyomas have been performed using fresh frozen tissue. Archival formalin-fixed paraffin-embedded (FFPE) tissue represents an invaluable source of biological material that can be studied retrospectively. Methods capable of generating high-quality data from FFPE material are in high demand. Here, we show that 3′RNA sequencing can accurately classify leiomyomas that have been stored as FFPE tissue in hospital archives for years. A targeted 3′RNA sequencing panel could provide researchers and clinicians with a cost-effective and scalable diagnostic tool for classifying smooth muscle tumors.

**Abstract:**

Uterine leiomyomas are benign smooth muscle tumors occurring in 70% of women of reproductive age. The majority of leiomyomas harbor one of three well-established genetic changes: a hotspot mutation in *MED12*, overexpression of *HMGA2*, or biallelic loss of *FH*. The majority of studies have classified leiomyomas by complex and costly methods, such as whole-genome sequencing, or by combining multiple traditional methods, such as immunohistochemistry and Sanger sequencing. The type of specimens and the amount of resources available often determine the choice. A more universal, cost-effective, and scalable method for classifying leiomyomas is needed. The aim of this study was to evaluate whether RNA sequencing can accurately classify formalin-fixed paraffin-embedded (FFPE) leiomyomas. We performed 3′RNA sequencing with 44 leiomyoma and 5 myometrium FFPE samples, revealing that the samples clustered according to the mutation status of *MED12*, *HMGA2*, and *FH*. Furthermore, we confirmed each subtype in a publicly available fresh frozen dataset. These results indicate that a targeted 3′RNA sequencing panel could serve as a cost-effective and robust tool for stratifying both fresh frozen and FFPE leiomyomas. This study also highlights 3′RNA sequencing as a promising method for studying the abundance of unexploited tissue material that is routinely stored in hospital archives.

## 1. Introduction

Uterine leiomyomas, or fibroids, are benign tumors originating from the smooth muscle cells of the myometrium. Leiomyomas are one of the most common human neoplasms, affecting 70% of women of reproductive age [1]. Although considered benign, leiomyomas frequently cause a variety of symptoms including pressure upon adjacent organs, abnormal uterine bleeding, pelvic pain, and impaired fertility [2]. Leiomyomas are the leading indication for hysterectomy worldwide and pose a significant socio-economic impact [3]. Approximately 10% of leiomyomas display nonconventional histopathology or abnormal growth patterns, some of which have been associated with specific molecular features [4].

Recent studies have revealed the existence of various molecular leiomyoma subtypes [5]. Indeed, 80−90% of leiomyomas harbor one of three genetic changes: a hotspot mutation in mediator complex subunit 12 (*MED12*), a chromosomal aberration resulting in significant overexpression of high mobility group AT-hook 2 (*HMGA2*), or biallelic loss of fumarate hydratase (*FH*). Leiomyomas with FH deficiency constitutes a rare but clinically relevant subtype, as pathogenic germline variants in *FH* predispose to hereditary leiomyomatosis and renal cell cancer (HLRCC). This syndrome is characterized by an early onset of multiple symptomatic uterine leiomyomas as well as renal cell cancer and cutaneous leiomyomas [6]. The majority of FH-deficient uterine leiomyomas are, however, sporadic. Other less frequent aberrations have been reported in leiomyomas, but most of these have been detected as subclonal alterations that co-occur with the other established driver aberrations [5]. For example, high mobility group AT-hook 1 (*HMGA1*) and PLAG1 zinc finger (*PLAG1*) rearrangements have been detected in both tumors with and without a *MED12* mutation [7,8,9]. Early observations suggest that different molecular subtypes behave differently, in terms of both clinical outcomes and therapeutic requirements [6,10,11]. For example, leiomyomas of the *MED12* subtype are typically more numerous, smaller in size, and subserosal [11]; leiomyomas of the *HMGA2* subtype are larger, isolated, and have a high vasculature density making them potentially sensitive to angiogenesis inhibitors [6,10]. Leiomyomas of the *FH* subtype have been associated with variant histopathology, including some histologic features associated with malignancy [6].

While the strongest evidence of each molecular subtype may be derived from whole-genome sequencing, it is not always the most feasible and cost-effective method for classifying leiomyomas. Most studies have therefore utilized cytogenetics, immunohistochemistry, qPCR, and Sanger sequencing [12,13,14]. Indeed, Sanger sequencing is the most common method for detecting hotspot mutations in exons 1 and 2 of *MED12*, whereas cytogenetics, immunohistochemistry, and qPCR have all been utilized for detecting *HMGA2* aberrations. Biallelic loss of *FH* can be accurately detected by immunohistochemistry with an antibody against S-(2-succinyl)cysteine, which is a chemical modification generated by high levels of fumarate [15].

We and others have previously shown that *MED12*, *HMGA2*, and *FH* aberrations result in distinct global gene expression patterns, suggesting that leiomyomas can be classified by gene expression profiling [7,14]. However, the majority of molecular studies on leiomyomas have been performed using fresh frozen tissue. While fresh frozen tissue might be ideal for most molecular analyses, archival formalin-fixed paraffin-embedded (FFPE) tissue represents an invaluable source of biological material that is routinely stored in hospital archives [16]. FFPE tissue has many advantages, including long-term storage at room temperature, abundance and availability, and connection to valuable clinical data such as histopathology and patient outcomes. However, FFPE-derived RNA is degraded and chemically modified, limiting its use in molecular studies [17]. Methods capable of generating high-quality transcriptome data from archival FFPE tissues are in high demand [16].

RNA sequencing has become an essential tool in cancer research, drug development, and cancer diagnostics [18]. One bias of standard whole-transcript RNA sequencing is that longer transcripts are represented by more reads than shorter transcripts [19]. Many recent RNA sequencing technologies, such as TagSeq and QuantSeq, have been developed to minimize this bias [20,21]. These techniques capture and sequence short fragments at the 3′ end of poly-adenylated RNA, generating only one read per transcript. 3′RNA sequencing has low RNA input and quality requirements, making it suitable for use with FFPE samples. Unlike gene expression microarrays, these newer technologies have the ability to utilize low amounts of RNA to generate transcriptome data that are comparable between FFPE and fresh frozen samples [22]. In this study, we have evaluated the potential of 3′RNA sequencing in analyzing archival leiomyoma material. In addition, we have assessed whether a set of 80 previously reported gene expression biomarkers could function as a targeted RNA sequencing panel to classify both fresh frozen and FFPE leiomyomas.

## 2. Results

### 2.1. Principal Component Analysis Confirms Different Molecular Subtypes

**Figure 1:**
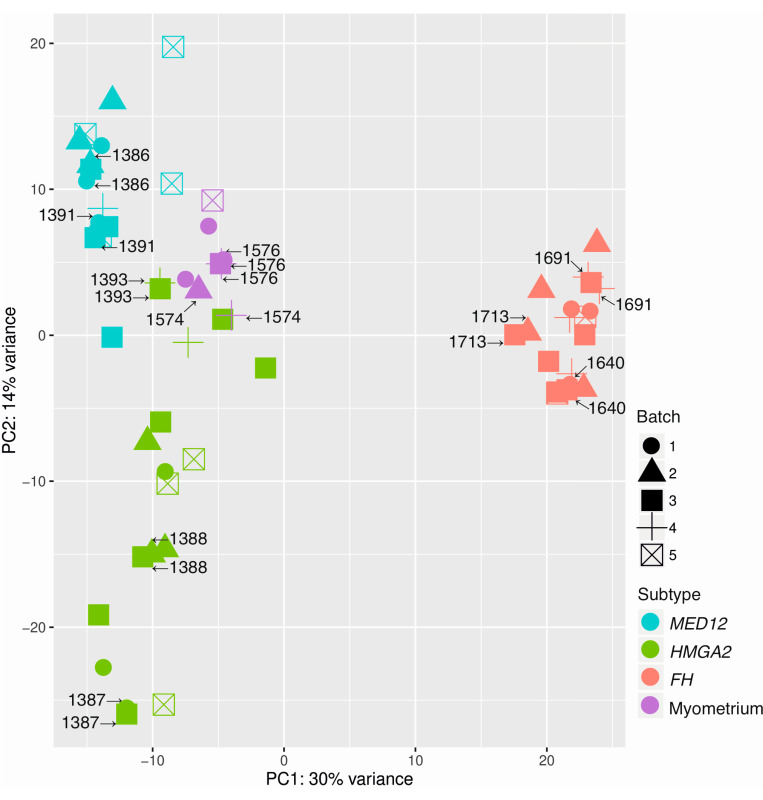
Principal component analysis shows a high degree of reproducibility. Principal component analysis using all genes revealed high reproducibility and no batch effect among the 49 samples and 11 technical replicates. The technical replicates clustered next to each other and the samples grouped according to the mutation status of *MED12*, *HMGA2*, and *FH*.

Principal component analysis using the 3′RNA sequencing data revealed that the 49 (44 leiomyomas + 5 myometrium) samples and 11 technical replicates grouped according to the predetermined mutation status of *MED12*, *HMGA2*, and *FH* (Figure 1). The age of the tissue material ranged from 4 to 31 years. The samples were sequenced in five batches, and the dataset included eleven technical replicates. We found a weak negative relationship (r = −0.27) between the age of the block and total read counts (Table S1). Taken together, these observations show high reproducibility and no obvious batch effect (Figure 1).

### 2.2. Differential Expression Analysis Confirms Distinct Gene Expression Patterns

**Figure 2:**
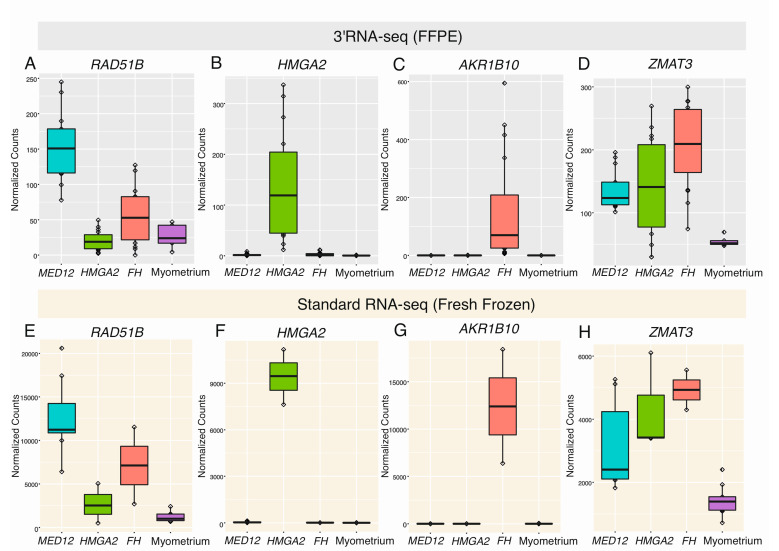
Highlighted genes previously shown to be dysregulated in leiomyomas. (**A**) Using 3′RNA sequencing of formalin-fixed paraffin-embedded (FFPE) tissue, we confirmed significant upregulation of *RAD51B* in leiomyomas with a *MED12* mutation. (**B**) We also confirmed significant upregulation of *HMGA2* in leiomyomas displaying positive IHC staining for *HMGA2*. (**C**) High expression levels of *AKR1B10* were only seen in leiomyomas displaying positive IHC staining for 2SC. (**D**) Upregulation of *ZMAT3* was seen in the majority of leiomyomas, regardless of subtype. (**E**–**H**) Similar observations were seen in an independent dataset of fresh frozen tissue that was sequenced using standard RNA sequencing.

We compared the 44 leiomyomas against the 5 myometrium samples, revealing 328 differentially expressed genes (*q* < 0.05; |FC| > 2, Table S2). We also compared each leiomyoma subtype against the myometrium samples, revealing 671 differentially expressed genes in leiomyomas of the *MED12* subtype, 905 in leiomyomas of the *HMGA2* subtype, and 1537 in leiomyomas of the *FH* subtype (*q* < 0.05; |FC| > 2, Table S2). See Figure S1 for a Venn diagram summarizing the number of shared and uniquely expressed genes among the different subtypes. We confirmed the expression pattern of several previously highlighted biomarkers (Figure 2A–D), including upregulation of RAD51 paralog B (*RAD51B*) in leiomyomas with a *MED12* mutation (*q*-value = 6.20 × 10^−13^, FC = 4.76), upregulation of *HMGA2* in leiomyomas with positive staining for HMGA2 (*q*-value = 4.84 × 10^−19^, FC = 28.44), upregulation of aldo-keto reductase family 1 member B10 (*AKR1B10*) in leiomyomas with positive staining for 2SC (*q* = 1.54 × 10^−07^, FC = 11.47), and upregulation of zinc finger matrin-type 3 (*ZMAT3*) in all leiomyoma subtypes (*q*-value = 2.90 × 10^−04^, FC = 2.69). The same expression pattern was also seen in an independent and publicly available fresh frozen dataset of 13 leiomyoma and 9 matching myometrium samples that were sequenced using standard RNA sequencing (Figure 2E–H). However, high expression of *AKR1B10* was observed in two fresh frozen samples that were originally classified as *HMGA1* overexpressing leiomyomas (Figure 2G).

### 2.3. Supervised Hierarchical Clustering Accurately Classifies Leiomyomas

**Figure 3:**
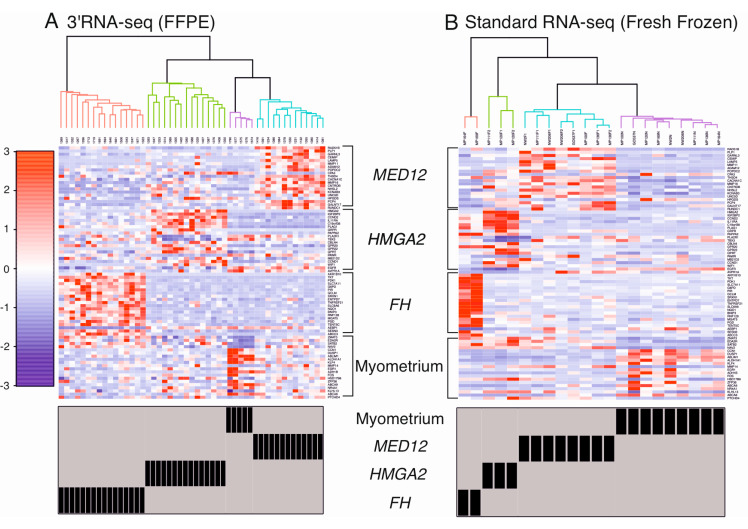
Supervised hierarchical clustering analysis using a set of 80 selected genes. (**A**) Hierarchical clustering analysis revealed that the 49 FFPE samples clustered into four distinct groups, representing the three most common leiomyoma subtypes and myometrium controls. (**B**) The same clustering analysis was performed with an independent dataset of 22 fresh frozen samples that were sequenced using standard RNA sequencing [14], revealing four similar groups. Two of the fresh frozen samples, originally classified as *HMGA1* overexpressing leiomyomas (MP164F, MP169F), displayed expression patterns of leiomyomas of the *FH* subtype.

Supervised hierarchical clustering analysis using a set of 80 genes previously shown to be dysregulated in leiomyomas revealed that the samples clearly clustered into four distinct groups, representing the three most common leiomyoma subtypes and the normal myometrium controls (Figure 3A). The same clustering analysis was performed with the fresh frozen dataset. Again, the samples clustered clearly into four similar groups, and the two *HMGA1* overexpressing leiomyomas displayed clear expression patterns of leiomyomas of the *FH* subtype (Figure 3B).

### 2.4. Exome Sequencing Reveals Biallelic Loss of FH in Two HMGA1 Overexpressing Leiomyomas

**Figure 4:**
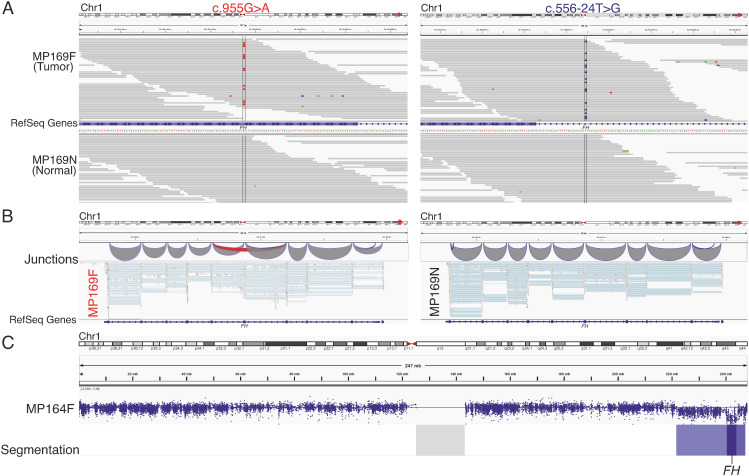
Biallelic loss of *FH* in two samples originally classified as *HMGA1* overexpressing tumors. (**A**) One *HMGA1* overexpressing tumor (MP169F) harbored a somatic missense variant c.955G > A, p.(Asp319Asn) in *FH* and a somatic substitution c.556-24T > G in intron 4 of *FH*. (**B**) The intronic variant resulted in RNA transcripts lacking exon 5. (**C**) Copy number analysis revealed a homozygous deletion encompassing *FH* in the other sample (MP164F).

Two samples, originally classified as *HMGA1* overexpressing leiomyomas [14], displayed clear expression patterns of leiomyomas of the *FH* subtype. Exome sequencing revealed a somatic missense variant *FH* c.955G > A, p.(Asp319Asn) and a somatic intronic variant *FH* c.556-24T > G in one of the samples (MP169F, Figure 4A). The missense variant was predicted deleterious with a Combined Annotation-Dependent Depletion (CADD) score of 28 [23], and the intronic variant was predicted to result in loss of a natural splice acceptor site according to spliceAI with a delta score of 0.56 [24]. Indeed, the intronic variant resulted in skipping of exon 5 according to the RNA sequencing data (Figure 4B). Both of the mutations were also present in the RNA sequencing data of the tumor and absent in the normal myometrium. Copy number analysis of the exome sequencing data revealed a homozygous deletion of *FH* in the other sample (MP164F, Figure 4C).

### 2.5. HMGA1 and PLAG1 Are Upregulated in Leiomyomas of the FH Subtype

**Figure 5:**
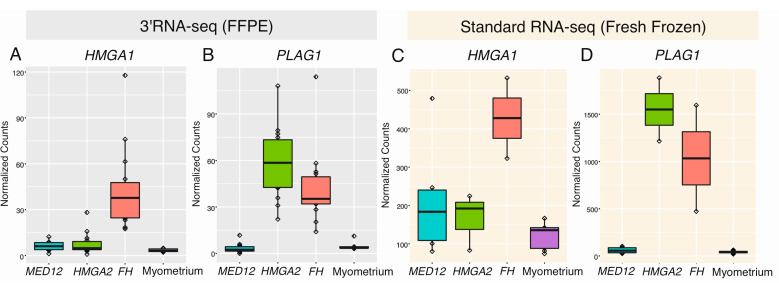
Expression of *HMGA1* and *PLAG1* in different leiomyoma subtypes and myometrium samples. (**A**,**C**) In both the FFPE and fresh frozen datasets, significant upregulation of *HMGA1* was only seen in the leiomyomas of the *FH* subtype, (**B**,**D**) whereas significant upregulation of *PLAG1* was seen in both leiomyomas of the *HMGA2* and *FH* subtypes, but not in leiomyomas of the *MED12* subtype nor the myometrium samples.

As we detected biallelic loss of *FH* in two leiomyomas previously reported to overexpress *HMGA1*, we explored the expression pattern of *HMGA1* in our dataset as well. We also explored the expression pattern of *PLAG1* in both datasets, since both HMGA1 and HMGA2 have been proposed to regulate the expression of this gene [7,25]. *HMGA1* was significantly upregulated in only leiomyomas of the *FH* subtype (*q*-value = 5.64 × 10^−14^, FC = 9.00), whereas *PLAG1* was significantly upregulated in both leiomyomas of the *HMGA2* (*q*-value = 4.84 × 10^−19^, FC = 9.13) and *FH* subtypes (*q*-value = 7.81 × 10^−14^, FC = 6.45) (Figure 5).

## 3. Discussion

Uterine leiomyomas are benign smooth muscle tumors that are still widely regarded as a single entity despite well-known heterogeneity in histology and emerging evidence of potential differences in clinical characteristics and response to therapies. Recent studies have also revealed that the majority of leiomyomas can be classified into three main molecular subtypes, reflecting mutations in *MED12*, *HMGA2*, and *FH*. Furthermore, we and others have shown that each subtype is characterized by a distinct global gene expression pattern [7,14], suggesting that leiomyomas could be classified by gene expression profiling. However, RNA-based studies on leiomyomas have largely been limited to fresh frozen tissue specimens. Archival FFPE tissue represents an invaluable source of biological material that could be used for retrospective studies. Using 3′RNA sequencing, we sequenced 49 FFPE samples in multiple batches and included 11 technical replicates, revealing high reproducibility and no noticeable batch effect. We also confirmed several previously reported subtype-specific patterns in two independent datasets and demonstrated that 3′RNA sequencing can accurately classify FFPE leiomyomas. This method of sequencing is relatively cost-effective and can be performed with low quantities of tissue material. Indeed, our samples were sequenced with only 80 ng of RNA and included FFPE material that was as old as 31 years.

We have previously highlighted a set of dysregulated genes in leiomyomas that could assist in their classification [7]. Here, we confirm that FFPE leiomyomas can similarly be classified into the three most common subtypes using the 80 most significant of these genes. In addition, we confirmed the accuracy of these 80 genes in a second independent dataset comprising fresh frozen samples that were sequenced using standard RNA sequencing. Furthermore, this led to the identification of biallelic loss of *FH* in two tumors that were originally classified as *HMGA1* overexpressing tumors by George et al. [14]. This motivated us to explore the expression pattern of *HMGA1* in our FFPE dataset, revealing significant upregulation of *HMGA1* in leiomyomas of the *FH* subtype, but not in leiomyomas of the *MED12* and *HMGA2* subtypes. *HMGA2* has been shown to directly regulate the expression of the oncogene *PLAG1*, suggesting that the homologue *HMGA1* may have a similar role in tumorigenesis [7]. As expected, we detected significant upregulation of *PLAG1* in leiomyomas of the *HMGA2* and *FH* subtypes, but not in leiomyomas of the *MED12* subtype. It is tempting to speculate that leiomyomas of the *FH* subtype may in part promote tumorigenesis through dysregulation of *HMGA1*.

There are also some limitations of the study. While all of the samples in both datasets clustered accurately according to the mutation status, the sample size is still relatively small. In addition, although the great majority of leiomyomas can be explained by defects in *MED12*, *HMGA2*, or *FH*, there is a small subset of leiomyomas where the driver alteration is unknown. We do not expect the inclusion of such samples to have a strong effect on the clustering, but this needs to be confirmed in further studies with larger sample sizes. Possible challenges related to clinical translation include the requirement of bioinformatics knowledge and the time required for data production and analysis.

Taken together, these observations demonstrate that our set of gene expression biomarkers are highly effective in classifying leiomyomas, regardless of whether the samples are fresh frozen or FFPE. These results also highlight 3′RNA sequencing as a promising method for studying the abundance of tissue material that is routinely stored in hospital archives.

## 4. Materials and Methods

### 4.1. Study Material and Sample Selection

The research has been approved by The Ethics Review Board of Hospital District of Helsinki and Uusimaa, Helsinki, Finland (ethical codes: 88/13/03/03/2015 and 24/13/03/03/2015). All samples were collected with the authorization from the National Supervisory Authority for Welfare and Health (Valvira) or with a written informed consent from the patients. The study material consisted of 49 archival FFPE tissue samples and corresponding hematoxylin-eosin stained slides that were obtained from the Department of Pathology, Helsinki University Hospital, Helsinki, Finland. Histopathological re-examination of the tumor and normal tissue slides was performed by a pathologist (RB or AP). The age of the tissue material ranged from 4 to 31 years. The mutation statuses of *MED12*, *HMGA2*, and *FH* were determined in five previously published studies [13,26,27,28,29]. See Table S3 for an overview of the mutation status of each sample. In brief, the mutation status of *MED12* was determined by Sanger sequencing, and the overexpression of *HMGA2* and the loss of *FH* were determined by immunohistochemistry using antibodies against HMGA2 and 2SC, respectively. The 49 FFPE samples comprised 13 leiomyomas with a *MED12* mutation, 15 with significant overexpression of HMGA2, 16 with FH deficiency, and 5 myometrium samples that served as normal tissue controls. The samples entered 3′RNA sequencing in 5 batches and included 11 technical replicates from 10 samples. In addition, we analyzed publicly available sequencing data of 13 fresh frozen leiomyomas and 9 corresponding myometrium samples. The tumors in this dataset were originally classified by George et al. [14] as 8 leiomyomas with a *MED12* mutation, 3 with significant overexpression of *HMGA2*, and 2 with significant overexpression of *HMGA1*.

### 4.2. RNA Extraction and Sequencing

Total RNA was extracted from 2 to 4 macrodissected 10 um tissue sections. The RNA yields ranged from 2175−41,940 ng with an average yield of 11,070 ng. RNA was extracted and purified using the RNeasy^®^ FFPE Kit (QIAGEN, Hilden, Germany) and the deparaffinization solution (QIAGEN) according to the manufacturer’s protocol. The concentration and purity of the extracted RNA were analyzed using the LabChip GX Touch HT RNA Assay Reagent Kit (PerkinElmer, Waltham, MA, USA) and the Qubit RNA BR kit (Thermo Fisher Scientific, Waltham, MA, USA). Genomic DNA contamination was measured using the Qubit DNA BR kit (Thermo Fisher Scientific). DNase treatment was performed to eliminate genomic DNA fragments.

Dual-indexed mRNA libraries were prepared from 80 ng of total RNA with QuantSeq 3′mRNA-Seq Library Prep Kit FWD (Lexogen Gmbh, Vienna, Austria) according to the manufacturer’s instructions. During second strand synthesis, 6 bp unique molecular identifiers (UMI) were introduced with the UMI Second Strand Synthesis Module (Lexogen Gmbh) for detection and removal of PCR duplicates. The quality of the libraries was measured with LabChip GX Touch HT DNA High Sensitivity Reagent Kit (PerkinElmer). The libraries were multiplexed and sequenced using the NovaSeq 6000 System (Illumina, San Diego, CA, USA) at the Institute for Molecular Medicine Finland (FIMM) with a read length of 2 × 101 base pairs and a minimum target coverage of 15 M reads for each library. Total number of aligned reads obtained for each sample are listed in (Table S1).

### 4.3. RNA Sequencing Data Analysis

FASTQ preprocessing was performed with default parameters using the QuantSeq 3′mRNA-Seq Integrated Data Analysis Pipeline version 2.3.1 FWD UMI (Lexogen Gmbh) implemented on the Bluebee^®^ Genomics platform. In brief, the reads were trimmed using BBDuk, aligned against the Genome Reference Consortium human build 38 (GRCh38) reference genome using Spliced Transcripts Alignment to a Reference (STAR) [30], and counted using HTSeq [31]. Principal component analysis (PCA) and differential expression analysis were performed using DESeq2 [32] implemented on the Chipster platform [33]. As no batch effect was observed in the PCA analysis, the read counts of the technical replicates were merged for the subsequent analyses. Additional raw RNA sequencing data (FASTQ) of 13 leiomyoma and 9 corresponding myometrium samples from the discovery dataset of George et al. [14] were retrieved from the Sequence Read Archive (SRA) database (BioProject PRJNA498292). The samples were aligned against the GRCh38 reference genome using STAR [34], and the reads were counted using HTSeq [31].

Raw read counts were normalized using DESeq2. Supervised hierarchical clustering analysis using Spearman’s rank correlation was performed with 80 genes previously shown to be differentially expressed in leiomyomas (Table S4) [6]. These 80 genes represent the 20 most uniquely expressed genes in leiomyomas of the *MED12*, *HMGA2*, and *FH* subtypes as well as the 20 most significantly differentially expressed genes in normal myometrium compared to all leiomyomas (*q* < 0.05; |FC| > 2).

### 4.4. Whole-Exome Sequencing

Raw whole-exome sequencing (WES) data (FASTQ) of two *HMGA1* overexpressing leiomyomas and eight myometrium samples from the discovery dataset of George et al. [14] were retrieved from the Sequence Read Archive (SRA) database (BioProject PRJNA494823). Adapter and read trimming were performed using Trimmomatic [35]. Data preprocessing was performed according to Genome Analysis Toolkit 4 best practices [36]. In brief, the samples were aligned against the GRCh38 reference genome using Burrows-Wheeler Aligner (BWA-MEM) [37], duplicate reads were removed using Mark Duplicates, and base quality score recalibration was performed using BaseRecalibrator. Somatic variant calling was performed against the corresponding normal using Mutect2 [38]. Somatic variants affecting the exonic and intronic regions of *FH* were evaluated further using BasePlayer [39]. Somatic copy number alterations were called using CNVkit against a pooled reference generated with the eight myometrium samples [40].

## 5. Conclusions

This proof-of-principle study demonstrates that 3′RNA sequencing is highly suitable for analyzing and classifying archival smooth muscle tissue material. Future work utilizing this method will not only provide insights into underlying defects of the remaining uncharacterized leiomyomas, but also allow for detection of biomarkers that can differentiate leiomyomas from their malignant counterparts: leiomyosarcomas. 3′RNA sequencing may provide researchers with an accurate tool to retrospectively study the transcriptome of rare tumor subtypes and cancers that are difficult to obtain. To conclude, our observations indicate that a targeted 3′RNA sequencing panel could provide researchers and clinicians with a cost-effective and scalable diagnostic tool for stratifying smooth muscle tumors. Millions of women suffer from leiomyomas, and the ability to accurately stratify each lesion should pave the way towards personalized treatments.

## Supplementary Materials

The following are available online at https://www.mdpi.com/2072-6694/12/12/3839/s1, Figure S1: Venn diagram demonstrating shared and uniquely expressed genes in leiomyomas of the different subtypes. Table S1: Age of the samples and number of reads generated by RNA sequencing. Table S2: Differentially expressed genes in each leiomyoma subtype. Table S3: Mutation status of uterine leiomyoma samples used in 3′ RNA sequencing. Table S4: A set of 80 significantly dysregulated genes in uterine leiomyomas.
