# Supplementary material for: 3′RNA Sequencing Accurately Classifies Formalin-Fixed Paraffin-Embedded Uterine Leiomyomas
Source: Cancers (Basel). 2020 Dec 19;12(12):3839. doi: 10.3390/cancers12123839 (PMC7766537; doi:10.3390/cancers12123839)
Supplement: Supplementary file 1 [file cancers-12-03839-s001.zip › cancers-1001905_supplementary_conversion/cancers-1001905_Supplementary_conversion.docx]

Supplementary Material


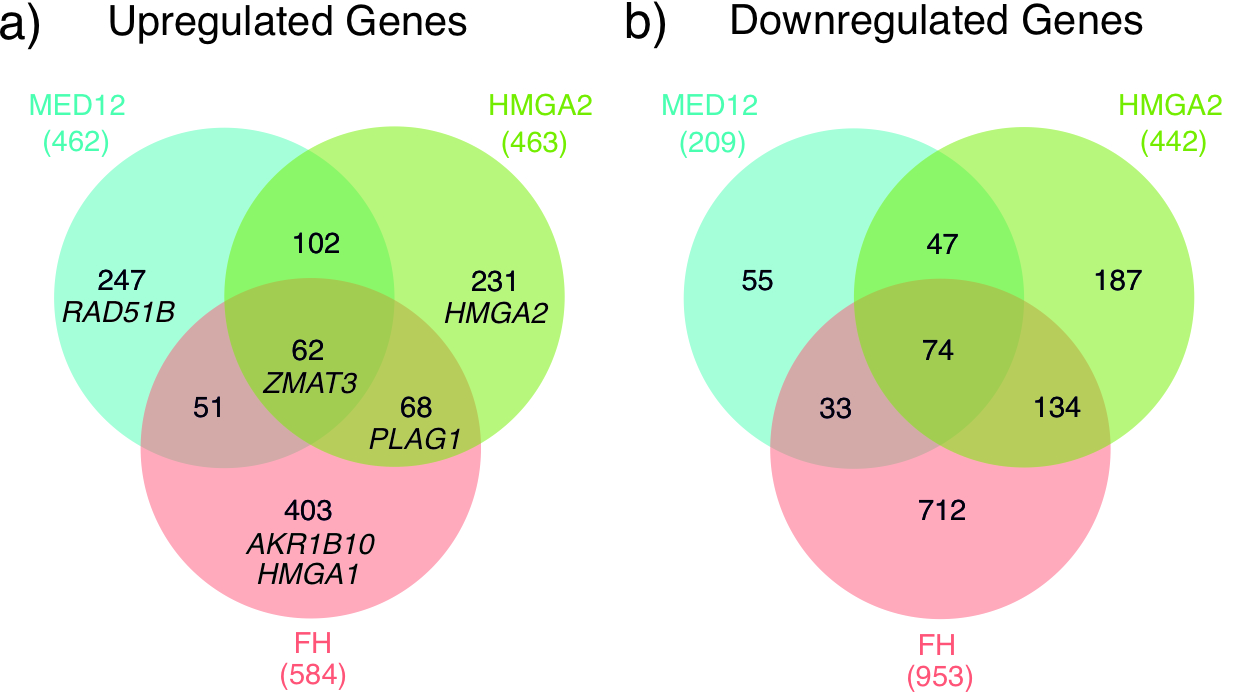


**Figure S1.** Venn diagram demonstrating shared and uniquely expressed genes in leiomyomas of the different subtypes. Comparing each leiomyoma subtype against the myometrium samples revealed 671 differentially expressed genes in leiomyomas of the *MED12* subtype, 905 in leiomyomas of the *HMGA2* subtype, and 1537 in leiomyomas of the *FH* subtype (*q*<0.05; −2>FC>2). We confirmed previously observed gene expression patterns in the genes *RAD51B*, *HMGA2*, *AKR1B10*, and *ZMAT3*. We also detected significant upregulation of *HMGA1* in leiomyomas of the *FH* subtype, whereas *PLAG1* was significantly upregulated in both leiomyomas of the *HMGA2* and *FH* subtypes.**Table S1.** Age of the samples and number of reads generated by RNA sequencing.

| **ID** | **Batch** | **Age of sample** | **Total aligned reads** | **Method** |
| --- | --- | --- | --- | --- |
| 1323_1_S1 | 5 | 4 | 5625999 | 3'RNAseq |
| 1369_1_S1 | 5 | 4 | 258932 | 3'RNAseq |
| 1790_2_S10 | 5 | 4 | 822017 | 3'RNAseq |
| 1355_1_S1 | 5 | 5 | 2550249 | 3'RNAseq |
| 1350_1_S1 | 5 | 5 | 1867308 | 3'RNAseq |
| 1671_1_S1 | 1 | 6 | 1021393 | 3'RNAseq |
| 1506_1_S1 | 1 | 6 | 3158955 | 3'RNAseq |
| 1343_1_S1 | 2 | 6 | 662928 | 3'RNAseq |
| 1332_1_S1 | 2 | 7 | 1202787 | 3'RNAseq |
| 1574_1_S1 | 2 | 7 | 3078053 | 3'RNAseq |
| 1574_1_S1 | 4 | 7 | 2203715 | 3'RNAseq |
| 1575_1_S1 | 1 | 7 | 2097132 | 3'RNAseq |
| 1576_1_S1 | 1 | 7 | 1469924 | 3'RNAseq |
| 1576_1_S1 | 3 | 7 | 1223175 | 3'RNAseq |
| 1576_1_S1 | 4 | 7 | 1443944 | 3'RNAseq |
| 1330_1_S1 | 5 | 8 | 1850908 | 3'RNAseq |
| 1329_1_S1 | 3 | 8 | 2297073 | 3'RNAseq |
| 1719_1_S1 | 2 | 8 | 538995 | 3'RNAseq |
| 1577_1_S1 | 1 | 8 | 3468299 | 3'RNAseq |
| 1386_1_S1 | 2 | 9 | 2230166 | 3'RNAseq |
| 1386_1_S1 | 3 | 9 | 2874047 | 3'RNAseq |
| 1416_1_S1 | 3 | 9 | 5573717 | 3'RNAseq |
| 1380_1_S1 | 2 | 9 | 1146812 | 3'RNAseq |
| 1414_1_S1 | 3 | 9 | 4196830 | 3'RNAseq |
| 1391_1_S1 | 1 | 9 | 4230613 | 3'RNAseq |
| 1391_1_S1 | 3 | 9 | 4256737 | 3'RNAseq |
| 1378_1_S1 | 2 | 9 | 1079514 | 3'RNAseq |
| 1381_1_S1 | 1 | 9 | 1660916 | 3'RNAseq |
| 1384_1_S1 | 1 | 9 | 2144279 | 3'RNAseq |
| 1047_3_S4 | 5 | 9 | 2598736 | 3'RNAseq |
| 1376_1_S1 | 4 | 9 | 1275621 | 3'RNAseq |
| 1387_1_S1 | 1 | 9 | 1660916 | 3'RNAseq |
| 1387_1_S1 | 3 | 9 | 4711614 | 3'RNAseq |
| 1388_1_S1 | 2 | 9 | 2651005 | 3'RNAseq |
| 1388_1_S1 | 3 | 9 | 3935053 | 3'RNAseq |
| 1389_1_S1 | 1 | 9 | 2310444 | 3'RNAseq |
| 1392_1_S1 | 2 | 9 | 842812 | 3'RNAseq |
| 1393_1_S1 | 3 | 9 | 1367922 | 3'RNAseq |
| 1393_1_S1 | 4 | 9 | 1749153 | 3'RNAseq |
| 1402_1_S1 | 2 | 9 | 1856519 | 3'RNAseq |
| 1407_1_S1 | 1 | 9 | 2624359 | 3'RNAseq |
| 1408_1_S1 | 3 | 9 | 1909768 | 3'RNAseq |
| 1640_1_S1 | 1 | 9 | 1314238 | 3'RNAseq |
| 1640_1_S1 | 3 | 9 | 2339770 | 3'RNAseq |
| 1713_1_S1 | 2 | 9 | 614566 | 3'RNAseq |
| 1713_1_S1 | 3 | 9 | 1672632 | 3'RNAseq |
| 1132_1_S2 | 4 | 10 | 1292365 | 3'RNAseq |
| 1317_1_S1 | 4 | 10 | 584549 | 3'RNAseq |
| 1691_1_S1 | 3 | 10 | 1606998 | 3'RNAseq |
| 1691_1_S1 | 4 | 10 | 2494866 | 3'RNAseq |
| 1314_1_S1 | 5 | 11 | 1171702 | 3'RNAseq |
| 1694_1_S1 | 4 | 11 | 3043264 | 3'RNAseq |
| 1253_1_S1 | 3 | 12 | 713089 | 3'RNAseq |
| 1305_1_S1 | 3 | 15 | 2512923 | 3'RNAseq |
| 1247_1_S1 | 4 | 21 | 580210 | 3'RNAseq |
| 1921_1_S3 | 3 | 22 | 1561556 | 3'RNAseq |
| 1231_1_S1 | 5 | 24 | 1225867 | 3'RNAseq |
| 1266_1_S1 | 3 | 24 | 563653 | 3'RNAseq |
| 1267_1_S1 | 3 | 28 | 601536 | 3'RNAseq |
| 1234_1_S1 | 5 | 31 | 828033 | 3'RNAseq |
| NW206F2 | NA | NA | 49981102 | Standard RNAseq |
| NW206N | NA | NA | 53214587 | Standard RNAseq |
| NW206F1 | NA | NA | 54720764 | Standard RNAseq |
| NW2F1 | NA | NA | 56528353 | Standard RNAseq |
| NW2N | NA | NA | 55641455 | Standard RNAseq |
| MP164F | NA | NA | 52033877 | Standard RNAseq |
| MP164N | NA | NA | 24495158 | Standard RNAseq |
| MP120N | NA | NA | 62082048 | Standard RNAseq |
| MP120F1 | NA | NA | 63719111 | Standard RNAseq |
| MP120F2 | NA | NA | 62223123 | Standard RNAseq |
| MP169N | NA | NA | 56299549 | Standard RNAseq |
| MP169F | NA | NA | 53020272 | Standard RNAseq |
| MP136N | NA | NA | 54020764 | Standard RNAseq |
| MP136F1 | NA | NA | 49729677 | Standard RNAseq |
| MP136F2 | NA | NA | 45461327 | Standard RNAseq |
| GO537N | NA | NA | 50295216 | Standard RNAseq |
| GO537F1 | NA | NA | 52189273 | Standard RNAseq |
| MP111N | NA | NA | 56316172 | Standard RNAseq |
| MP111F1 | NA | NA | 55841486 | Standard RNAseq |
| MP111F2 | NA | NA | 52387159 | Standard RNAseq |
| MP100N | NA | NA | 58945966 | Standard RNAseq |
| MP100F | NA | NA | 61255399 | Standard RNAseq |

**Table S3.** Mutation status of uterine leiomyoma samples used in 3’RNA sequencing.

| **Sample ID** | **Tissue** | ***MED12* mutation [ref.]** | **HMGA2 [ref.]** | **FH (2SC) [ref.]** |
| --- | --- | --- | --- | --- |
| 1386_1_S1 | Leiomyoma | c.100-22_102del [1] | NEG [2] | NEG [3] |
| 1416_1_S1 | Leiomyoma | c.107T>G, p.(Leu36Arg) [1] | NEG [2] | NEG [3] |
| 1380_1_S1 | Leiomyoma | c.117_134del, p.(Leu39_Gly44del) [1] | NEG [2] | NEG [3] |
| 1414_1_S1 | Leiomyoma | c.122T>A, p.(Val41Glu) [1] | NEG [2] | NEG [3] |
| 1231_1_S1 | Leiomyoma | c.126_134del, p.(Lys42_Phe45delinsAsn) [1] | NEG [2] | NEG [2] |
| 1132_1_S2 | Leiomyoma | c.130G>A, p.(Gly44Ser) [4] | NEG [4] | NEG [4] |
| 1234_1_S1 | Leiomyoma | c.130G>T, p.(Gly44Cys) [1] | NEG [2] | NEG [2] |
| 1391_1_S1 | Leiomyoma | c.130G>T, p.(Gly44Cys) [1] | NEG [2] | NEG [3] |
| 1378_1_S1 | Leiomyoma | c.131G>A, p.(Gly44Asp) [1] | NEG [2] | NEG [3] |
| 1381_1_S1 | Leiomyoma | c.131G>A, p.(Gly44Asp) [1] | NEG [2] | NEG [3] |
| 1384_1_S1 | Leiomyoma | c.131G>A, p.(Gly44Asp) [1] | NEG [2] | NEG [3] |
| 1330_1_S1 | Leiomyoma | c.131G>A, p.(Gly44Asp) [5] | NEG [5] | NEG [5] |
| 1355_1_S1 | Leiomyoma | c.131G>C, p.(Gly44Ala) [5] | NEG [5] | NEG [5] |
| 1253_1_S1 | Leiomyoma | wt [1] | POS [2] | NEG [2] |
| 1305_1_S1 | Leiomyoma | wt [1] | POS [2] | NEG [2] |
| 1047_3_S4 | Leiomyoma | wt [1] | POS [2] | NEG [2] |
| 1314_1_S1 | Leiomyoma | wt [1] | POS [2] | NEG [2] |
| 1376_1_S1 | Leiomyoma | wt [1] | POS [2] | NEG [3] |
| 1387_1_S1 | Leiomyoma | wt [1] | POS [2] | NEG [3] |
| 1388_1_S1 | Leiomyoma | wt [1] | POS [2] | NEG [3] |
| 1389_1_S1 | Leiomyoma | wt [1] | POS [2] | NEG [3] |
| 1392_1_S1 | Leiomyoma | wt [1] | POS [2] | NEG [3] |
| 1393_1_S1 | Leiomyoma | wt [1] | POS [2] | NEG [3] |
| 1402_1_S1 | Leiomyoma | wt [1] | POS [2] | NEG [3] |
| 1407_1_S1 | Leiomyoma | wt [1] | POS [2] | NEG [3] |
| 1408_1_S1 | Leiomyoma | wt [1] | POS [2] | NEG [3] |
| 1329_1_S1 | Leiomyoma | wt [5] | POS [5] | NEG [5] |
| 1350_1_S1 | Leiomyoma | wt [5] | POS [5] | NEG [5] |
| 1267_1_S1 | Leiomyoma | wt [1] | NEG [2] | POS [2] |
| 1247_1_S1 | Leiomyoma | wt [1] | NEG [2] | POS [2] |
| 1317_1_S1 | Leiomyoma | wt [1] | NEG [2] | POS [2] |
| 1266_1_S1 | Leiomyoma | wt [1] | NEG [2] | POS [2] |
| 1921_1_S3 | Leiomyoma | wt [3] | NEG [This study] | POS [3] |
| 1640_1_S1 | Leiomyoma | wt [4] | NEG [4] | POS [4] |
| 1671_1_S1 | Leiomyoma | wt [4] | NEG [4] | POS [4] |
| 1691_1_S1 | Leiomyoma | wt [4] | NEG [4] | POS [4] |
| 1694_1_S1 | Leiomyoma | wt [4] | NEG [4] | POS [4] |
| 1713_1_S1 | Leiomyoma | wt [4] | NEG [4] | POS [4] |
| 1719_1_S1 | Leiomyoma | wt [4] | NEG [4] | POS [4] |
| 1506_1_S1 | Leiomyoma | wt [4] | NEG [4] | POS [4] |
| 1343_1_S1 | Leiomyoma | wt [5] | NEG [5] | POS [5] |
| 1332_1_S1 | Leiomyoma | wt [5] | NEG [5] | POS [5] |
| 1323_1_S1 | Leiomyoma | wt [5] | NEG [5] | POS [5] |
| 1369_1_S1 | Leiomyoma | wt [5] | NEG [5] | POS [5] |
| 1790_2_S10 | Myometrium | NA | NA | NA |
| 1574_1_S1 | Myometrium | NA | NA | NA |
| 1575_1_S1 | Myometrium | NA | NA | NA |
| 1576_1_S1 | Myometrium | NA | NA | NA |
| 1577_1_S1 | Myometrium | NA | NA | NA |

**Table S4.** A set of 80 significantly dysregulated genes in uterine leiomyomas.

| ***MED12*** | ***HMGA2*** | ***FH*** | **Myometrium/** |
| --- | --- | --- | --- |
|  |  |  | **All leiomyomas** |
| *RAD51B*  *PLP1* | *HMGA2*  *IGF2BP2* | *AKR1B10* | *ZMAT3* |
|  |  | *TKT* | *EDA2R* |
| *GARNL3* | *CCND2* | *PDK1* | *SATB2* |
| *CEMIP* (*KIAA1199*) | *IL11RA* | *SLC7A11* | *NAV2* |
| *LAMP5* | *C19orf38* | *G6PD* | *CCN1* (*CYR61*) |
| *MMP11* | *PLAG1* | *PIR* | *DUSP1* |
| *ADAM12* | *GRPR* | *GCLM* | *ABLIM1* |
| *POPDC2* | *PAPPA2* | *SRXN1* | *ALDH1A1* |
| *CPA3* | *PLA2R1* | *ENTPD7* | *KLF4* |
| *THSD4* | *TBX3* | *TNFRSF21* | *MMP14* |
| *CACNA1C* | *CBLN4* | *SLC6A6* | *EGR1* |
| *MMP16* | *GPR20* | *NQO1* | *ADH1B* |
| *CNTROB* | *GPR22* | *BNIP3* | *FOS* |
| *NHSL2* | *QPRT* | *RNF128* | *HSD17B6* |
| *KCNAB3* | *PAWR* | *MGAT5* | *ZFP36* |
| *UNC5D* | *MB21D2* | *PGD* | *ABCA9* |
| *HPGDS* | *CCND1* | *TENT5C* (*FAM46C*) | *NR4A1* |
| *PCP4* | *WIF1* | *AEBP1* | *ABCA6* |
| *GALNT17* (*WBSCR17*) | *EGFR* | *SESN3* | *KLHL13* |
| *RUNDC1* | *AVPR1A* | *ABCC3* | *PTCHD4* |

References

1. Mäkinen, N.; Vahteristo, P.; Kämpjärvi, K.; Arola, J.; Butzow, R.; Aaltonen, L.A. MED12 exon 2 mutations in histopathological uterine leiomyoma variants. *Eur J Hum Genet* **2013***, 21*, 1300-1303.
2. Mäkinen, N.; Kämpjärvi, K.; Frizzell, N.; Butzow, R.; Vahteristo, P. Characterization of MED12, HMGA2, and FH alterations reveals molecular variability in uterine smooth muscle tumors. *Molecular cancer* **2017***, 16*, 101-101.
3. Kämpjärvi, K.; Mäkinen, N.; Mehine, M.; Välipakka, S.; Uimari, O.; Pitkänen, E.; Heinonen, H.; Heikkinen, T.; Tolvanen, J.; Ahtikoski, A.; Frizzell, N.; Sarvilinna, N.; Sjöberg, J.; Bützow, R.; Aaltonen, L.A.; Vahteristo, P. MED12 mutations and FH inactivation are mutually exclusive in uterine leiomyomas. *Br J Cancer* **2016**. 1405-1411.
4. Äyräväinen, A.; Pasanen, A.; Ahvenainen, T.; Heikkinen, T.; Pakarinen, P.; Härkki, P.; Vahteristo, P. Systematic molecular and clinical analysis of uterine leiomyomas from fertile-aged women undergoing myomectomy. *Hum Reprod* **2020***, 35*, 2237-2244.
5. Ahvenainen, T.V.; Mäkinen, N.M.; von Nandelstadh, P.; Vahteristo, M.E.A.; Pasanen, A.M.; Bützow, R.C.; Vahteristo, P.M. Loss of ATRX/DAXX expression and alternative lengthening of telomeres in uterine leiomyomas. *Cancer* **2018***, 124*, 4650-4656.

| 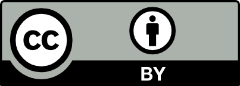 | © 2020 by the authors. Submitted for possible open access publication under the terms and conditions of the Creative Commons Attribution (CC BY) license (http://creativecommons.org/licenses/by/4.0/). |
| --- | --- |
